# Round versus tape sutures with interference fit anchors perform similarly in cadaveric glenoid and greater tuberosity biomechanical testing

**DOI:** 10.1007/s00402-026-06524-9

**Published:** 2026-10-07

**Authors:** James D Dimitroff, Conor Smith, Stefan Hanish, Lasun O. Oladeji, Will Bezold, Clayton W Nuelle, Matthew J Smith

**Affiliations:** 1 Department of Orthopaedic Surgery, University of Missouri, Columbia, USA; 2 Orthopedic Surgery at the Mercy Center for Performance Medicine, Mercy Hospital, St Louis, USA; 3 Campbell Clinic, Memphis, USA; 4 Department of Orthopaedic Surgery, University of Miami, Coral Gables, USA; 5 Thompson Laboratory for Regenerative Orthopedics, Columbia, USA

**Keywords:** Shoulder biomechanics, Rotator cuff repair, Labral repair, Cadaveric study, Arthroscopic shoulder surgery

## Abstract

**Purpose:**

To determine whether suture shape (flat-tape vs. round-wire) influences biomechanical performance at the suture-anchor–bone interface in interference-fit anchors used for rotator cuff and glenoid labrum repair.

**Methods:**

Four matched pairs of human cadaveric shoulders were used for the glenoid model, with six anchor sites per glenoid. Five matched pairs of cadaveric humeri were used for the rotator cuff model, with three anchor sites per humerus. Each model tested multiple suture-anchor constructs under cyclic loading followed by load-to-failure. Biomechanical outcomes included cyclic displacement, yield characteristics, ultimate failure strength, stiffness, and force at fixed displacements. A linear mixed-effects model was used to compare suture types, with suture type and anchor position as fixed effects and specimen as a random effect.

**Results:**

In the glenoid/labrum model, there were no significant differences among suture types in cyclic displacement (cycles 1, 50, or 100; *p* = 0.376, 0.101, 0.109), displacement at yield (*p* = 0.406), yield force (*p* = 0.880), force at 1 mm (*p* = 0.264) or 5 mm (*p* = 0.085), or ultimate failure strength (*p* = 0.204). Stiffness (*p* = 0.050) and force at 2 mm displacement (*p* = 0.033) differed at the overall model level, however, no significant pairwise differences were observed. In the humerus/rotator cuff model, displacement at cycle 50 (*p* = 0.022) and cycle 100 (*p* = 0.019) differed overall, although post hoc comparisons were not significant. Ultimate strength (*p* = 0.037), yield force (*p* = 0.012), and stiffness (*p* = 0.005) differed among groups, with post hoc analysis showing greater ultimate strength, yield force, and stiffness for 2-mm FiberTape compared with 2-mm XbraidTT. No other pairwise comparisons were significant.

**Conclusion:**

Suture shape did not meaningfully influence interference-fit fixation properties in the glenoid and humeral models evaluated in this study. Tape-type and round-type sutures demonstrated comparable biomechanical performance at the suture–anchor interface within the tested constructs.

**Level of evidence:**

Controlled Laboratory Study.

## Introduction

Rotator cuff tears are the leading cause of shoulder pain in middle-aged to elderly populations, resulting in over 500,000 surgeries annually in the United States, with nearly 90% now performed arthroscopically [1–3]. The high prevalence of arthroscopic RCR, in part, reflects the application of biomechanical research to refine surgical technique, allowing surgeons to optimize critical aspects of repair including suture-to-bone fixation, suture-to-tendon fixation, suture abrasion resistance, suture strength, knot security, and loop security [4]. 

A key consideration in this optimization is suture selection. The two primary suture designs are the traditional interwoven round-type wire suture and a broader, flat tape-type suture. Tape-type sutures have demonstrated favorable characteristics at the suture-tendon interface, including a wider contact area, increased footprint contact pressures, and up to 31% higher load to failure. [5–6] Biomechanical studies have also shown that although tape-type sutures create a larger initial hole when passed through the tendon, standard tape-type sutures enlarge their initially small holes and exhibit greater displacement during cyclic loading [7]. This suggests that tape-type sutures may reduce the risk of pullout at the suture-tendon interface; however, clinical studies have not demonstrated a significant difference in retear rates between the two designs [6–8]. 

While these prior studies have primarily focused on the suture-tendon interface, there is currently no evidence directly comparing the biomechanical performance of different suture shapes, round versus flat, within interference-fit anchors at the suture-anchor interface. This question is particularly relevant given that interference-fit anchors achieve fixation by compressing the suture against the bone within the anchor body and are commonly used in arthroscopic rotator cuff repairs [9]. Although clinical evidence indicates that suture pullout is not a predominant mode of failure in RCR, biomechanical studies have demonstrated that the load required to cause suture slippage is frequently lower than the force required to dislodge the anchor from bone. [10–11] These findings indicate that suture slippage remains a plausible failure mode in interference-fit systems.

Additionally, the question of which suture shape is ideal in interference fit anchors also applies to other shoulder surgeries, such as labral repair (LR). While multiple studies have examined variables such as anchor composition and design, suture configuration, anchor placement and insertion angle, and trabecular bone quality in LR, none have investigated how suture shape affects suture pullout strength [12–16]. 

Therefore, the purpose of this study was to examine the biomechanical properties of various suture types in interference-fit anchors in both the greater tuberosity, as a model for RCR, and the glenoid, for LR. We hypothesized flat tape-type sutures, owing to their low-profile geometry when compressed beneath an interference-fit anchor, would be more susceptible to slippage and therefore demonstrate less suture pullout strength than round-type sutures at the suture-anchor interface in RCR and LR.

## Methods

### Cadaveric specimen preparation

Five pairs of fresh-frozen cadaveric shoulders (mean age, 44.4 years [range, 32–50 years]; three male and two female specimens) were obtained from a commercial source (Science Care, Phoenix, AZ, USA). Institutional review board approval was not required because the study used commercially obtained cadaveric specimens and was conducted in accordance with institutional policies governing the use of cadaveric tissue. The shoulders were stored at − 20 °C until thawed at room temperature for 24 h before dissection by fellowship trained orthopaedic surgeons. The proximal humerus was dissected free of all soft tissue. No significant abnormal anatomy or obvious evidence of poor bone quality was noted in any specimen. Objective measures of bone quality, such as bone mineral density, were not available.

#### Glenoid/Labrum

**Fig. 1:**
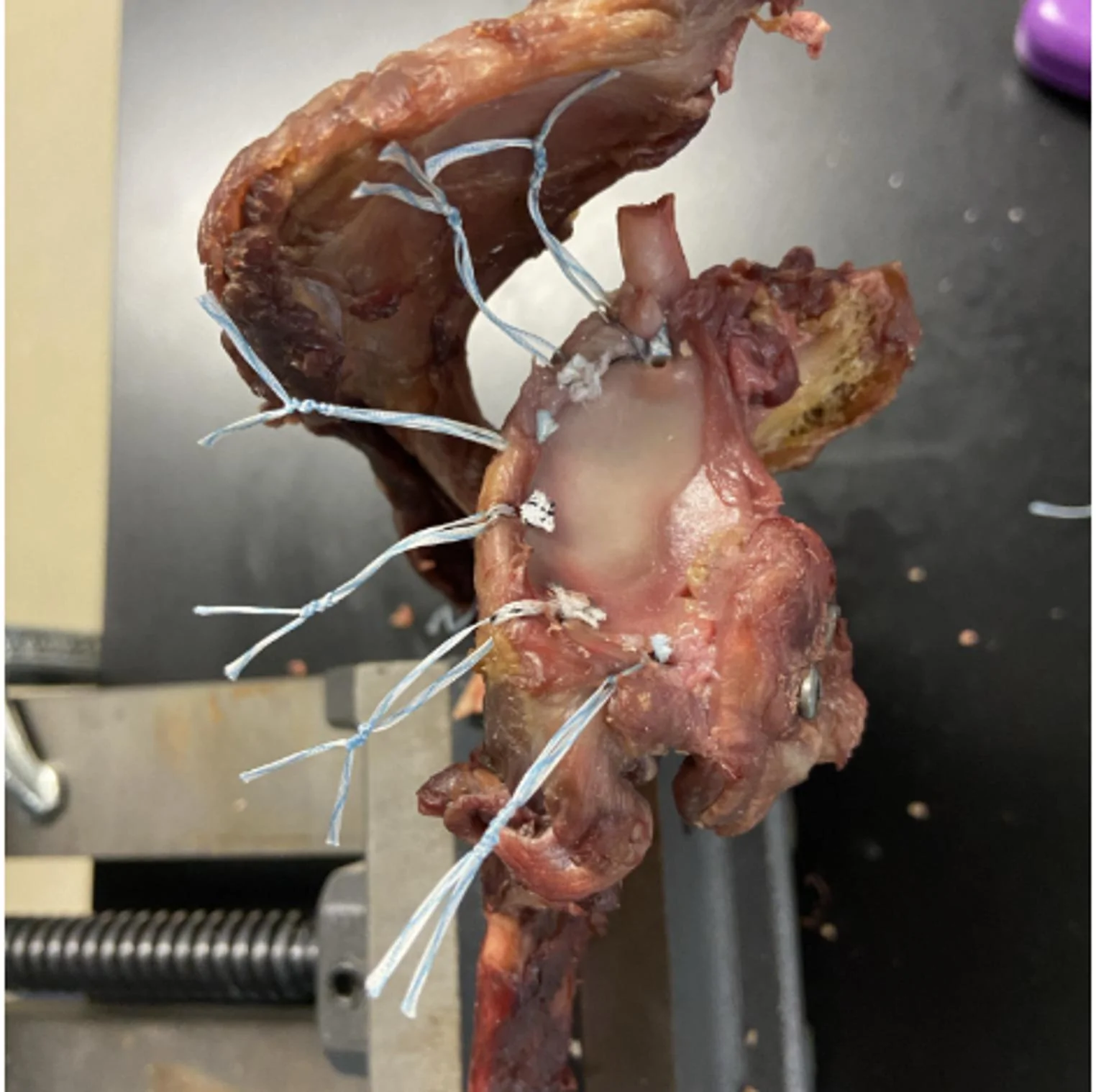
Representative glenoid specimen during preparation for biomechanical testing, demonstrating the six posterior anchor locations used in the present investigation. This specimen had previously undergone a postmortem Latarjet reconstruction; the retained coracoid graft and fixation screws visible anteriorly were removed before biomechanical testing

Four of the five glenoid pairs were utilized. One glenoid specimen pair was excluded before testing because of improper storage conditions that rendered it unsuitable for testing. The glenoid specimens were utilized across two planned biomechanical investigations to maximize the use of the cadaveric tissue while maintaining separate testing regions. As part of an unrelated investigation, the specimens had undergone standardized postmortem anterior glenoid reconstruction using either a Latarjet procedure or distal tibial allograft reconstruction, with two specimen pairs undergoing each procedure. The posterior glenoid was reserved for the present investigation because it remained separate from the anterior reconstruction and provided a suitable and reproducible location for standardized biomechanical testing. Before biomechanical testing for the present study, the prior anterior glenoid constructs and associated fixation hardware were removed. Anchor placement was limited to six standardized posterior glenoid locations, beginning at the 12 o’clock position and progressing posteriorly to the 6 o’clock position (Fig. 1), thereby avoiding the previously reconstructed anterior glenoid. Three suture-anchor combinations were tested: short 2.9-mm BioComposite PushLock anchors (Arthrex, Naples, FL, USA) with 1.3-mm SutureTape (A1.3), short 2.9-mm BioComposite PushLock anchors with 1.5-mm LabralTape (A1.5), and short 2.9-mm BioComposite PushLock anchors with #2 FiberWire (A2). Each suture construct was tested 16 times, for a total of 48 completed construct tests. The sutures were repaired over a second suture loop, facilitating connection to the Instron material testing machine **(**Instron, Norwood, MA). The location of the suture types alternated within each shoulder and staggered between shoulders to mitigate position on the glenoid as a confounding variable.

#### Humerus/Rotator cuff

**Fig. 2:**
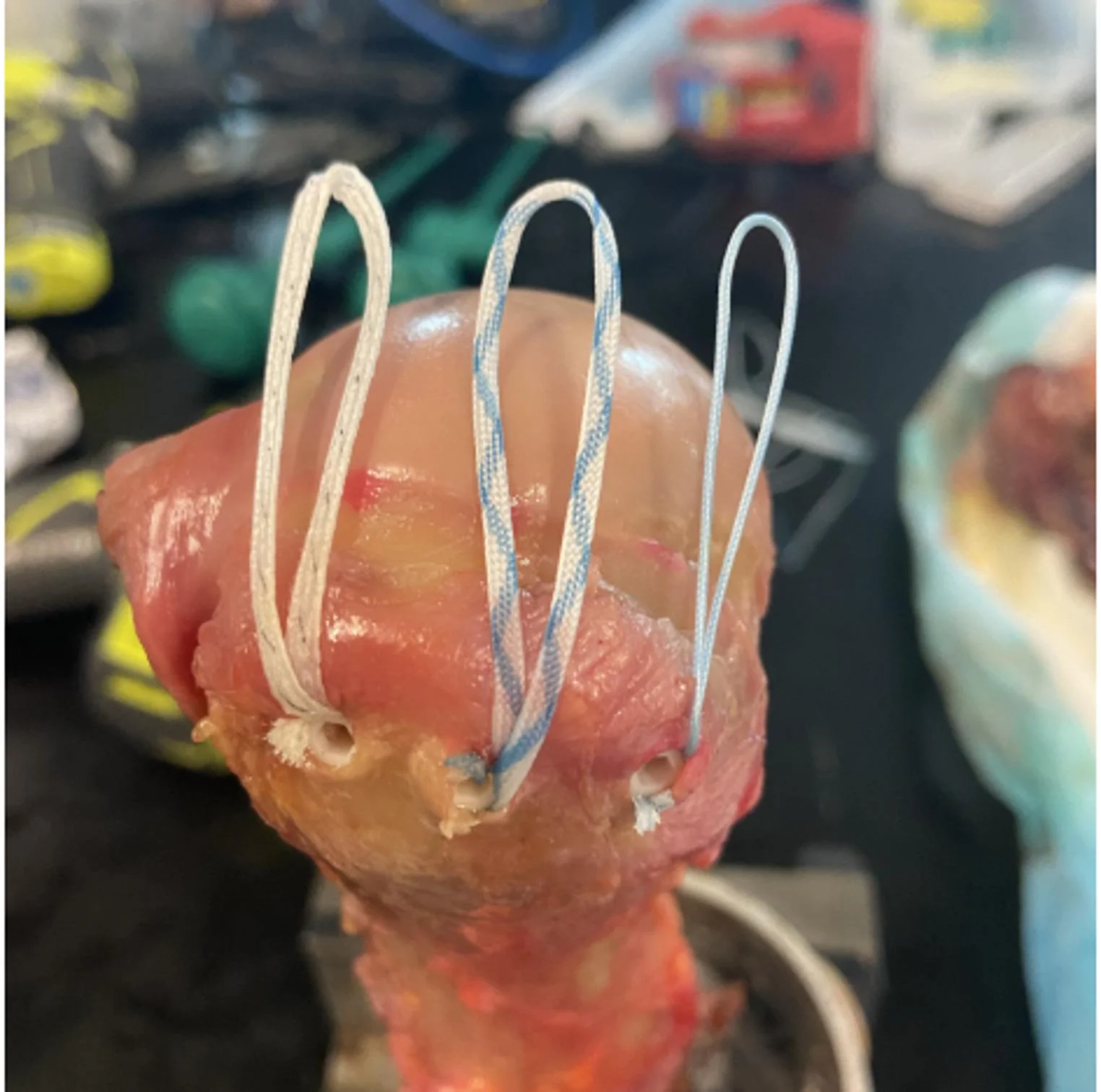
Proximal humerus specimen demonstrating the three suture anchor positions along the greater tuberosity footprint. From left to right: 2-mm FiberTape (AFT), 1.7-mm SutureTape (AST), and #2 FiberWire (A2)

All five humerus pairs were utilized. Each humerus had three discrete locations for anchor placement, starting at the 6 mm posterior to the lateral edge of the bicipital groove, spaced approximately 6 mm apart as shown in Fig. 2. Five suture-anchor type combinations were tested:

4.75-mm DX Knotless SwiveLock Suture Anchors (Arthrex, Naples, FL USA) with 1.7-mm flat SutureTape (AST), 4.75-mm SwiveLock with 2-mm flat FiberTape (AFT), 4.75-mm SwiveLock with #2 round FiberWire (A2), 4.75-mm Omega Self-Punching Knotless Anchor (Stryker, Kalamazoo, MI USA) with #2 round Force Fiber (SR), 4.75-mm Omega with 2-mm flat XBraid_TT_ (SF).

Thirty humeral tests were intentionally allocated to provide equivalent overall representation of each manufacturer’s constructs while ensuring that all constructs met or exceeded the a priori minimum sample size established by the power analysis. Because three Arthrex constructs and two Stryker constructs were evaluated, this resulted in sample sizes of five for AFT, five for AST, five for A2, seven for SR, and eight for SF. Sutures were tested using each manufacturer’s corresponding anchor system to avoid nonstandard construct combinations and better reflect clinically utilized repair constructs. Consequently, the independent effects of suture geometry and anchor design could not be completely separated. Suture construct locations were alternated within each humerus and staggered between shoulders to mitigate anchor position on the humerus as a confounding variable.

### Biomechanical testing

**Fig. 3:**
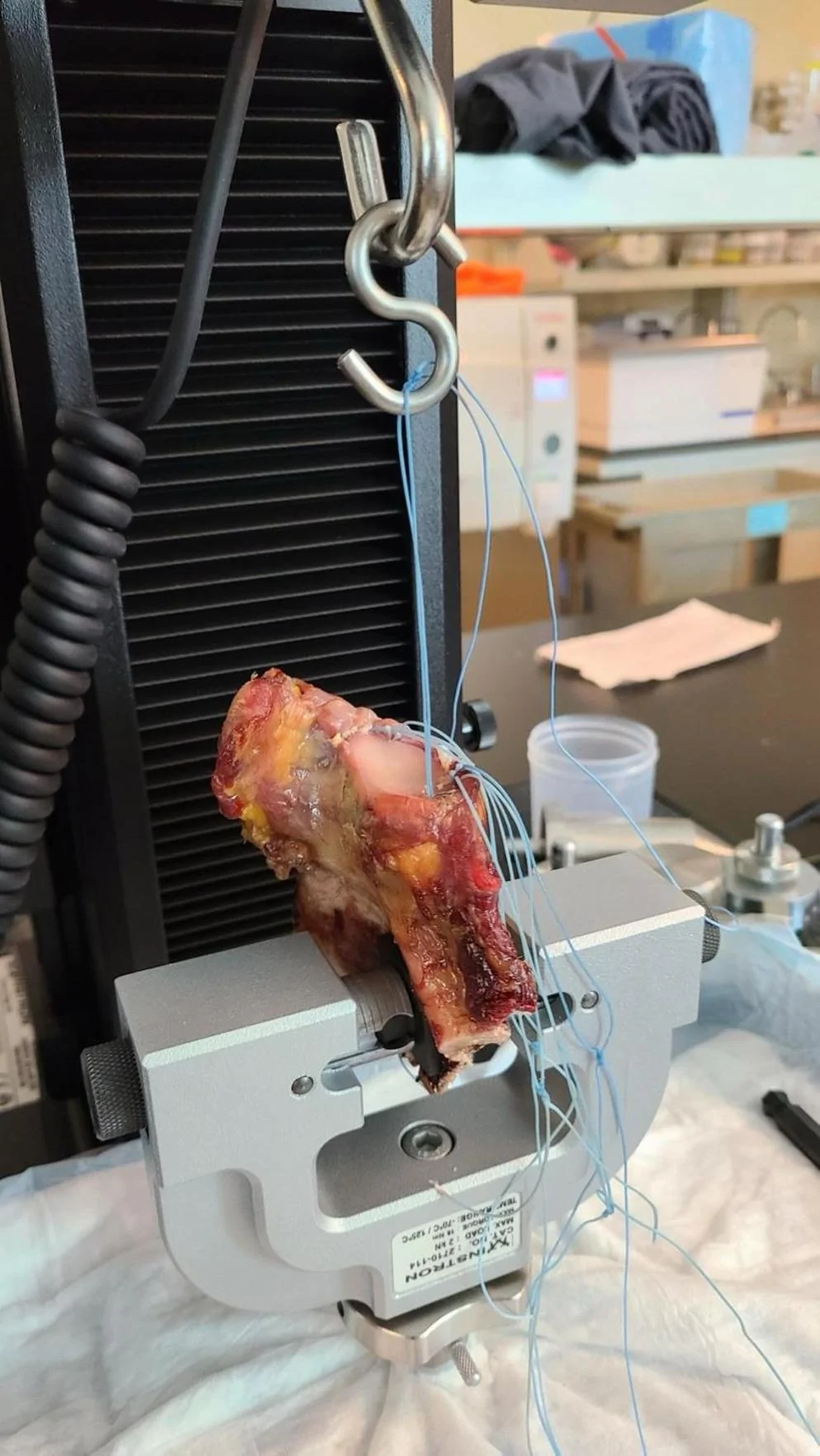
Glenoid specimen secured in a compression clamp on an Instron materials testing system following removal of the prior anterior glenoid reconstruction construct and associated fixation hardware

For the rotator cuff model, proximal humerus samples were potted into 25-mm-diameter copper tubes to facilitate biomechanical tests as shown in Fig. 2. The copper tube was inserted and fastened into a multi-position fixture that was rigidly attached to the table of an Instron material testing machine **(**Instron, Norwood, MA). For the glenoid labrum model, following removal of the prior anterior glenoid reconstruction construct and associated fixation hardware, the scapula was directly inserted and fastened into a compression clamp and rigidly attached to the table of an Instron material testing machine as shown in Fig. 3. The multi-position fixture allowed the specimen to be oriented such that the axis of the anchor was aligned in the direction of the axis of the loading machine. The ends of the suture for each anchor were attached to the crosshead of the test machine using an S-hook as seen in Fig. 3.

Each specimen underwent a standardized loading protocol consisting of preloading, cyclic loading, and load-to-failure testing. Specimens were first preloaded at 0.05 mm/s to 5 N and held at this load for 120 s to eliminate construct slack and allow viscoelastic equilibration, consistent with previously described biomechanical protocols [14, 17]. Cyclic loading was then performed between 10 and 50 N at 0.8 mm/s for 100 cycles. The 10–50 N loading range was selected based on established biomechanical protocols with comparable testing methodologies and was chosen to remain below the suture pullout threshold identified during pilot testing, thereby minimizing the risk of premature construct failure or suture slippage during cyclic loading [18, 19]. One hundred loading cycles were selected because prior biomechanical studies have demonstrated that the majority of construct displacement and settling occurs within the first 100 cycles [20, 21]. Following cyclic loading, specimens were loaded to failure at a displacement rate of 1 mm/s, a commonly used quasi-static loading rate that permits reliable identification of the construct yield point and ultimate failure.

Ultimate failure was observed to be pull out of the suture from the anchor in all cases; no failures occurred by anchor pullout from surrounding bone. Following failure of each anchor, the specimen was repositioned to allow subsequent testing of remaining anchors in a similar manner. The force and displacement were recorded in real time during each test using an acquisition rate of 50 Hz and a load cell of 100 kg. Throughout cyclic and LTF testing, load-displacement curves were generated to determine construct stiffness (N/mm) (defined as the slope of the load vs. displacement curve), force at yield point (N) (defined as the point in which the load vs. displacement curve deviated from linear), displacement at yield point (mm), ultimate failure strength (N) (the maximum load recorded), and force (N) at 1 mm, 2 mm, and 5 mm of failure.

#### Data analysis

Using pilot yield force data, an a priori one-way ANOVA power analysis was performed with yield force designated as the primary biomechanical endpoint. A minimum sample size of 5 specimens per suture-anchor construct was required based on a minimum difference in means of 15 N, a maximum standard deviation of 9 N, 80% power, and an α of 0.05. The one-way ANOVA was used as a practical approximation for sample-size estimation based on the planned between-group comparison of yield force. All statistical analyses were conducted in R (version 4.4.1; R Foundation for Statistical Computing, Vienna, Austria) with significance set at *P* < 0.05.

Because multiple anchor sites were tested within each cadaveric specimen, biomechanical measurements were not independent. Therefore, a linear mixed-effects model was used in place of one-way ANOVA to appropriately account for within-specimen correlation and natural variability in bone quality across donors, suture type and anchor position were treated as fixed effects and specimen was treated as a random effect. Group differences were summarized using estimated marginal means (EMMs), which provide model-adjusted mean values.

Mechanical testing trials with clearly identifiable procedural errors were excluded prior to analysis. Following model fitting, potential outliers were evaluated using standardized model residuals (|residual| > 3) and within-suture type Z-scores (|Z| > 3). Outlier candidates were removed only when statistical flagging corresponded to a visually identifiable mechanical testing artifact visible on the original force–displacement trace. Application of these predefined exclusion criteria resulted in the removal of six completed glenoid construct tests, yielding final analyzed sample sizes of 13 for A1.3, 14 for A1.5, and 15 for A2. No humeral construct tests met the predefined exclusion criteria.

Model assumptions were assessed using Q–Q plots (normality of residuals) and residuals-versus-fitted plots (homoscedasticity). These diagnostics demonstrated acceptable residual structure, supporting the appropriateness of the linear mixed-effects model without transformation.

Fixed effects were evaluated using a Type III analysis of variance with Satterthwaite’s approximation. When a significant main effect for suture type was present, Tukey-adjusted pairwise comparisons were performed. Because the omnibus Type III ANOVA evaluates the overall effect across all groups, significant omnibus findings may not correspond to statistically significant Tukey-adjusted pairwise comparisons after correction for multiple testing. Accordingly, conclusions regarding differences between individual suture constructs were based on the Tukey-adjusted pairwise comparisons, which control the family-wise error rate across all pairwise comparisons within each analysis. Statistical significance was defined as *P* < 0.05.

## Results

### Glenoid/Labrum

Biomechanical testing results for the glenoid labrum model are summarized in Table 1. There were no significant differences among suture types in displacement at cycles 1, 50, or 100 (*p* = 0.376, 0.101, and 0.109, respectively), or in ultimate failure strength (*p* = 0.204). Displacement at yield (*p* = 0.406), yield force (*p* = 0.880), force at 1 mm displacement (*p* = 0.264), and force at 5 mm displacement (*p* = 0.085) also did not differ significantly among groups. The Type III ANOVA on the mixed-effects model demonstrated significant differences in stiffness (*p* = 0.050) and force at 2 mm displacement (*p* = 0.033). However, Tukey-adjusted pairwise comparisons did not identify significant differences between individual suture types for either outcome.

**Table 1:** Model-adjusted biomechanical outcomes by suture type in the glenoid/labrum model

Outcome Data	A1.3	A1.5	A2	Position *p*-value	Suture *p*-value
Cycle 1 Displacement (mm)	4.4 ± 0.5	3.5 ± 0.5	4.3 ± 0.5	0.479	0.376
Cycle 50 Displacement (mm)	5.5 ± 0.6	4.7 ± 0.5	6.0 ± 0.5	0.470	0.101
Cycle 100 Displacement (mm)	5.7 ± 0.6	4.5 ± 0.5	5.8 ± 0.5	0.652	0.109
Ultimate Strength (N)	95.3 ± 8.6	112.1 ± 7.8	102.5 ± 8.2	0.781	0.204
Displacement at Yield (mm)	6.4 ± 0.8	5.5 ± 0.7	6.1 ± 0.7	0.674	0.406
Yield Force (N)	68.8 ± 8.1	72.4 ± 7.6	71.9 ± 7.9	0.966	0.880
Stiffness (N/mm)	13.8 ± 1.9	19.3 ± 1.7	15.4 ± 1.8	0.752	0.050*
Force at 1 mm (N)	3.1 ± 1.0	4.5 ± 0.9	3.8 ± 0.9	0.340	0.264
Force at 2 mm (N)	9.5 ± 4.0	18.7 ± 3.8	13.2 ± 3.9	0.252	0.033*
Force at 5 mm (N)	48.9 ± 5.5	62.2 ± 4.9	51.4 ± 5.2	0.542	0.085

Values are reported as model-adjusted means ± standard error (SE) from linear mixed-effects model. P-values correspond to the Type III ANOVA tests for the main effect of suture position and suture type. A1.3 = 1.3 mm SutureTape; A1.5 = 1.5 mm LabralTape; A2 = #2 FiberWire. Statistically significant comparisons are denoted by an asterisk (*; *P* < 0.05)

#### Humerus/Rotator cuff

Biomechanical outcomes for the humeral repair model are summarized in Table 2. There was no difference among suture types in displacement at cycle 1 (*p* = 0.064). Type III ANOVA on the mixed-effects model demonstrated significant differences in displacement at cycle 50 (*p* = 0.022) and cycle 100 (*p* = 0.019). Despite these overall effects, Tukey-adjusted pairwise comparisons did not identify significant differences between individual suture types for these displacement outcomes.

Force at 1 mm (*p* = 0.060), 2 mm (*p* = 0.097), and 5 mm (*p* = 0.114) of displacement, as well as displacement at yield (*p* = 0.113), did not differ significantly by suture type. In contrast, ultimate failure strength (*p* = 0.037), yield force (*p* = 0.012), and construct stiffness (*p* = 0.005) were significantly different across groups. Post hoc analysis identified that AFT sutures demonstrated greater ultimate failure strength (*p* = 0.042), higher yield force (*p* = 0.020), and greater stiffness (*p* = 0.008) compared with SF sutures. No other pairwise comparisons were statistically significant.

**Table 2:** Model-adjusted biomechanical outcomes by suture type in the humerus/rotator cuff model

Outcome Data	A2	AFT	AST	SF	SR	Position *p*-value	Suture *p*-value
Cycle 1 Displacement (mm)	2.9 ± 1.4	3.6 ± 1.4	4.9 ± 1.3	7.0 ± 1.0	4.7 ± 1.1	0.922	0.064
Cycle 50 Displacement (mm)	4.3 ± 1.6	4.2 ± 1.4	6.2 ± 1.4	8.6 ± 1.1	7.0 ± 1.2	0.882	0.022*
Cycle 100 Displacement (mm)	4.3 ± 1.7	4.3 ± 1.5	6.4 ± 1.4	8.9 ± 1.2	7.1 ± 1.2	0.877	0.019*
Ultimate Strength (N)	101.3 ± 16.9	147.7 ± 16.4	110.6 ± 15.8	96.5 ± 13.8	113.2 ± 14.3	0.814	0.037*
Displacement at Yield (mm)	5.6 ± 1.2	7.1 ± 1.1	6.7 ± 1.1	8.9 ± 0.9	7.2 ± 0.9	0.360	0.113
Yield Force (N)	78.6 ± 13.8	121.7 ± 13.3	83.7 ± 12.8	75.1 ± 11.1	81.6 ± 11.6	0.784	0.012*
Stiffness (N/mm)	15.9 ± 2.8	22.4 ± 2.6	13.9 ± 2.5	10.3 ± 2.0	13.4 ± 2.2	0.816	0.005*
Force at 1 mm (N)	3.8 ± 1.3	6.8 ± 1.2	6.1 ± 1.2	3.3 ± 1.0	4.7 ± 1.1	0.225	0.060
Force at 2 mm (N)	15.9 ± 3.9	21.6 ± 3.7	19.7 ± 3.5	11.5 ± 2.8	13.5 ± 3.3	0.377	0.097
Force at 5 mm (N)	61.5 ± 12.7	80.3 ± 12.1	55.9 ± 11.5	43.6 ± 9.2	56.4 ± 9.8	0.752	0.114

Values are reported as model-adjusted means ± standard error (SE) from linear mixed-effects model. P-values correspond to the Type III ANOVA tests for the main effect of suture position and suture type. A2 = #2 FiberWire; AFT = 2 mm FiberTape; AST = 1.7 mm SutureTape; SF = 2 mm XBraidTT; SR = #2 Force Fiber. Statistically significant comparisons are denoted by an asterisk (*; *P* < 0.05)

## Discussion

The hypothesis that round-type sutures would provide improved interference fit at the suture-anchor interface, thereby enhancing strength, stiffness, and displacement resistance, was not confirmed. In the glenoid/labrum model, no significant differences among suture types remained after pairwise comparison. In the humerus/rotator cuff repair model, Type III ANOVA demonstrated significant differences in five biomechanical parameters: Displacement at cycle 50 and 100, yield force, ultimate failure strength, and construct stiffness. However, post hoc comparisons demonstrated that only three of these variables (ultimate strength, yield force, and stiffness) differed significantly between any two suture types, and all these differences occurred exclusively between 2-mm FiberTape (AFT) and 2-mm XbraidTT (SF). Given that both are tape-type sutures and that all other statistical analyses were nonsignificant, our results indicate that suture shape alone does not meaningfully affect biomechanical performance between suture and bone in glenoid and humerus models.

Nevertheless, the observed differences between FiberTape (AFT) and XbraidTT may still help guide surgical decision-making. Suture selection remains largely driven by surgeon preference, which has increasingly favored suture tapes due to their favorable biomechanical performance at the most common point of failure, the suture-tendon interface [8]. However, no published studies to date have directly compared the biomechanical or clinical outcomes of different suture tapes. Because manufacturer-specific anchor systems were evaluated, the observed differences may reflect the combined effects of suture tape and anchor design rather than suture tape properties alone. Within this context, the FiberTape construct demonstrated greater stiffness and required higher force to pull out of the anchor than the XBraidTT construct. Although these findings should be interpreted cautiously, they may still help inform construct selection in clinical scenarios where higher initial fixation strength is desirable.

Most prior investigations have focused on the suture-tendon, although several studies have evaluated the intrinsic biomechanical properties of suture materials. Wright et al. [22] demonstrated that anchors loaded with polyethylene sutures FiberWire and Orthocord (Mitek, Somerville, NJ) exhibited higher load to failure and ultimate tensile strength compared with classic braided polyester sutures Ethibond (Ethicon, Somerville, NJ) and Tevdek (Deknatel, Mansfield, MA), and absorbable monofilament polydioxanone (PDS) suture in vitro. Several years later, Hurwit et al. [18] investigated the intrinsic viscoelastic properties of various ultrahigh–molecular-weight polyethylene (UHMWPE) sutures and found that FiberWire demonstrated the greatest stiffness and smallest peak-to-peak displacement when compared with MagnumWire (ArthroCare, Austin, TX), Ethibond, Orthocord, and Force Fiber (Tornier, Bloomington, MN). Building on this work, Taha et al. [19] compared the viscoelastic properties between wire-like and tape-like UHMWPE sutures, finding that tapes provided greater construct stiffness and a higher potential to minimize gap formation. These studies, however, were limited by their design, as sutures were tested in isolation on mechanical devices rather than within animal or human tissue.

In contrast to prior work assessing sutures in isolation, our findings suggest that intrinsic material differences do not necessarily translate into meaningful variations in performance at the suture-anchor interface. Favorable mechanical properties, such as stiffness, observed in isolation may be mitigated by the complex interactions between suture, anchor, and bone. Moreover, load distribution across the repair is influenced by multiple factors including bone quality, anchor design, and insertion angle, all of which may diminish the relative impact of suture shape alone. Collectively, these findings emphasize that optimizing repair biomechanics requires a system-level perspective that accounts for the integrated behavior of the entire construct.

Supporting this concept, Huntington et al. [23] compared the mechanical fatigue strength of suture wire and tape in an ovine model, reporting that tape demonstrated higher ultimate tensile strength than equivalent wire-constructs. Similarly, De Carli et al. [5], utilizing bovine specimens, found a 31% increase in ultimate tensile strength with suture-tape constructs compared with wire constructs. Despite these tensile advantages in animal models, our results did not reveal meaningful differences between tape and wire sutures when tested in human cadaveric bone at the suture-anchor interface. This discrepancy underscores the model-dependent nature of suture performance and emphasizes the importance of human tissue testing to better approximate clinically relevant behavior. Notably, however, both animal model studies reported no significant differences in stiffness or gap formation between tape-type and round-type sutures, consistent with our results.

This study evaluated biomechanical performance at the suture–anchor interface in human tissue models. By evaluating sutures within a system rather than in isolation, we believe that our findings more closely approximate the in vivo environment and provide insights that prior studies were unable to capture. However, given the greater complexity of our study design and evaluation of different biomechanical parameters, direct comparison with prior work is challenging. Because no significant differences were observed between tape-type and round-type sutures at the suture-anchor interface, the present data suggest that surgeons may not need to weigh suture performance at the suture-anchor interface when selecting their desired suture design. Instead, decision-making may be better directed toward addressing the most common complication observed in both biomechanical studies and clinical practice—failure at the suture-tendon interface [5, 6, 11, 23–25]. Future studies incorporating cyclic loading, varying bone quality, and in vivo animal or clinical models will be important to confirm these findings and further refine surgical decision-making.

## Limitations

 Institutional Review Board (IRB) approval was not required for this study as it was a biomechanical investigation restricted entirely to cadaveric specimens and did not involve living human participants or identifiable private information.The study was conducted in accordance with institutional guidelines for handling human tissue. First, the humeral model compared clinically utilized manufacturer-recommended suture-anchor constructs rather than nonstandard suture-anchor combinations. This design better reflects clinical practice but does not permit complete separation of the independent effects of suture geometry and anchor design. Therefore, differences observed between constructs may reflect the combined influence of both variables. Second, all glenoid specimens had undergone prior postmortem anterior glenoid reconstruction using either a Latarjet procedure or distal tibial allograft. Although the prior constructs were removed before testing and all anchors were placed at standardized posterior locations, the prior reconstruction may have altered glenoid architecture or bone quality and may limit the generalizability of these findings to native glenoid anatomy. Third, the constructs were tested under axial loading aligned with the anchor axis to provide a standardized and reproducible assessment of suture–anchor fixation. This loading configuration does not fully replicate the tangential forces experienced in vivo and therefore may not capture differences in suture–bone interaction or construct behavior under more physiologic loading conditions. Fourth, the use of cadaveric specimens restricts interpretation to time-zero biomechanics and does not directly inform the clinical implications of round versus flat suture selection, including biological healing or remodeling, repair durability, and patient outcomes following surgery. Fifth, while the loading protocol was designed to simulate physiological conditions, it cannot fully replicate the complex, multidirectional, and repetitive stresses experienced in vivo. Sixth, since the biomechanical properties of labral and rotator cuff tissue could change with age, BMI, and other factors, future studies could evaluate the impact of these variables on overall repair outcomes as it relates to variable anchor and suture type(s). Finally, the relatively limited sample size, while consistent with other biomechanical investigations, may reduce the ability to detect subtle differences between constructs.

## Conclusion

Suture shape did not meaningfully influence interference-fit fixation properties in the glenoid and humeral models evaluated in this study. Tape-type and round-type sutures demonstrated comparable biomechanical performance at the suture–anchor interface within the tested constructs.

## Data Availability

The datasets generated and analyzed during the current study are available from the corresponding author on reasonable request.
